# Enhancing insulin sensitivity in type 2 diabetes mellitus using apelin-loaded small extracellular vesicles from Wharton’s jelly-derived mesenchymal stem cells: a novel therapeutic approach

**DOI:** 10.1186/s13098-024-01332-w

**Published:** 2024-04-16

**Authors:** Jing Cui, Mingkun Wang, Wenhong Zhang, Jiachen Sun, Yan Zhang, Li Zhao, Zhibo Hong, Dongtao Li, Yi Xiong Huang, Ningkun Zhang, Yu Chen

**Affiliations:** 1 The Fifth School of Clinical Medicine, Navy Clinical College, Anhui Medical University, Hefei, Anhui China; 2 Department of Cardiology, The Sixth Medical Center of Chinese People’s Liberation Army General Hospital, Beijing, China· China; 3 Department of Dermatology, Peking University Third Hospital, Beijing, China

**Keywords:** Apelin, Type 2 diabetes mellitus, IR, Small extracellular vesicles, Wharton’s jelly-derived mesenchymal stem cells

## Abstract

**Background:**

Type 2 diabetes mellitus (T2DM), characterized by β-cell dysfunction and insulin resistance (IR), presents considerable treatment challenges. Apelin is an adipocyte-derived factor that shows promise in improving IR; however, it is limited by poor targeting and a short half-life. In the present study, engineered small extracellular vesicles (sEVs) derived from Wharton’s jelly-derived mesenchymal stem cells (WJ-MSCs) loaded with apelin were used to address the limitations of the therapeutic application of apelin.

**Methods:**

WJ-MSCs were transduced to obtain engineered sEVs loaded with overexpressed apelin (apelin-MSC-sEVs) and the control sEVs (MSC-sEVs). T2DM mice were injected with apelin-MSC-sEVs and MSC-sEVs, and blood glucose monitoring, glucose and insulin tolerance tests, confocal microscopy, and immunocytochemical analysis were performed. IR models of 3T3-L1 adipocytes were employed to detect GLUT4 expression in each group using western blotting; the affected pathways were determined by measuring the changes in Akt and AMPK signaling and phosphorylation.

**Results:**

Upon successful engineering, WJ-MSCs demonstrated significant overexpression of apelin. The genetic modification did not adversely impact the characteristics of sEVs, ranging from surface protein markers, morphology, to particle size, but generated apelin-overexpressed sEVs. Apelin-MSC-sEVs treatment resulted in notable enhancement of Akt and AMPK pathway activities within 3T3-L1 adipocytes and adipose tissues of T2DM mice. Furthermore, the apelin-loaded sEVs significantly reduced plasma glucose levels, increased pancreatic β-cell proliferation, improved insulin and glucose tolerance, and modulated pro-inflammatory cytokine profiles, compared to mice treated with the control sEVs.

**Conclusion:**

Our study developed novel genetically engineered apelin-loaded sEVs derived from WJ-MSCs, and demonstrated their potent role in augmenting insulin sensitivity and regulating inflammatory responses, highlighting their therapeutic promise in T2DM management. The findings open new avenues for the development of clinically viable treatments for T2DM in humans using the apelin-loaded sEVs.

**Supplementary Information:**

The online version contains supplementary material available at 10.1186/s13098-024-01332-w.

## Background

Type 2 diabetes mellitus (T2DM) is the most prevalent form of metabolic disorder; it is characterized by hyperglycemia and insulin resistance (IR) and exacerbated by high-fat diet-induced obesity, the most common cause of IR in humans [1, 2]. This pathological state, where the biological effects of insulin are diminished, underpins a variety of diseases, including diabetes, metabolic syndrome, and atherosclerotic cardiovascular diseases, manifesting as reduced insulin sensitivity in adipocytes, muscle cells, and hepatocytes, alongside the dysregulation of glucose, lipid, and protein metabolism [1, 3, 4]. Ultimately, this leads to the exacerbation of T2DM and subclinical systemic inflammation and chronic complications that affect the adipose tissue, liver, pancreas, hypothalamus, heart, and other tissues [5]. Current treatments primarily focus on insulin administration and chemical drugs, such as sulfonylureas, metformin, and thiazolidinediones, which temporarily manage blood glucose levels [6]. However, these approaches do not address the core issue of IR and may even lead to diminished insulin production and secretion by β-cells, thereby convoluting the mitigation of diabetic complications [6–8].

Adipocytes function as endocrine organs, secreting various adipokines that play key roles in regulating glucose homeostasis, insulin sensitivity, and the overall energy balance [9]. Apelin, a newly identified adipocyte-derived factor, acts as an endogenous ligand for the orphan G protein-coupled apelin receptor, which is essential for maintaining insulin sensitivity and resistance [10]. However, apelin has a biological half-life of less than 5 min before rapid clearance from the circulation, which significantly hinders its potential therapeutic application in reversing IR [11]. Our previous study demonstrated that Wharton’s jelly-derived mesenchymal stem cells (WJ-MSCs) transfected with apelin can promote rat pancreatic β-cell proliferation and maintain long-term glucose stability [12]. However, cell therapy has several limitations, including preservation challenges and stability issues [13]. Furthermore, the introduction of WJ-MSCs into the human body poses the potential risk of adverse effects from cell proliferation [14].

Small extracellular vesicles (sEVs) are tiny extracellular vesicular structures (approximately 40–160 nm in diameter) formed by the fusion of multivesicular bodies with the plasma membrane [15], and they have been shown to mimic the regenerative and anti-inflammatory functions of MSCs of origin [16]. Their ability to easily cross biological barriers as well as their low immunogenicity and phagocytosis resistance make them ideal carriers for delivering therapeutic agents into cells [17]. This study aimed to address the challenges associated with cell therapy by leveraging the unique biologic functions of engineered sEVs derived from MSCs loaded with apelin to create a novel biological nanomedicine, AP-MSC-sEVs. This approach not only circumvents the safety risks associated with direct stem cell transplantation but also avoids genetic alteration hazards, thus offering a synergistic solution to counteract IR. Furthermore, we sought to elucidate the molecular mechanisms underlying effects of apelin in reversing IR, providing a groundbreaking intervention for the pathological core mechanisms of various diseases.

## Methods

### Overexpression of Apelin in WJ-MSCs

WJ-MSCs were isolated as described previously [18]; cells collected from 3 to 4 generations were used. The study design was approved by the Ethics Review Committee of the Sixth Medical Center of the People’s Liberation Army in China. For further details, please refer to our previous publication [12].

Apelin-overexpressing plasmids were derived from an apelin-expressing lentiviral plasmid GV208-apelin with the lentiviral vector pReceiver-Lv203 (Genecopoeia, Rockville, MD, USA), which incorporates a CMV promoter, enhanced green fluorescent protein (eGFP), and the selection marker puromycin. The re-prepared pReceiver-Lv203-apelin clones were verified via DNA sequencing (GENEWIZ, China). To prepare the lentiviral particles, pReceiver-Lv203-apelin and pReceiver-Lv203 were used to transfect HEK 293T cells. Twenty hours post transfection, the medium was replaced with a fresh culture medium. After 48 h, the medium was collected and the virus was concentrated using a 100 KD ultrafiltration tube. Subsequently, the WJ-MSCs were transduced with the virus and selected for puromycin resistance to establish stable cell lines.

### Cell model

An IR model established using the 3T3-L1 cell line [19] was procured from ProCell and cultured in DMEM, containing 10% calf serum and 1% P/S (ProCell, China, CM-0006), in a humidified atmosphere at 37 °C with 5% CO_2_. To induce the differentiation of 3T3-L1 preadipocytes into adipocytes, the cells were seeded at an appropriate density in 6-well plates and cultured until they reached 70–80% confluency.

Two days post-confluence (day 0), cells were treated for two days with differentiation induction medium containing 0.5 mM 3-isobutyl-1-methylxanthine (Sigma Aldrich, Germany, I5879), 1 µM dexamethasone (Solarbio, China, D8040), and 10 µg/mL insulin (RHAWN, China, R008974). On day 2 of differentiation induction, the medium was replaced with DMEM containing 10 µg/mL insulin, along with 10% FBS and 1% PS. On day 4, the medium was replaced with DMEM containing 10% FBS. The cells were fed every two days with DMEM supplemented with 10% FBS to maintain normal growth.

### Purification, characterization, and uptake of sEVs derived from apelin-overexpressing WJ-MSCs

WJ-MSCs were cultured in a serum-free medium (Yocon, China, NC0106) as reported [12]. Upon achieving 70–90% confluence, the cell supernatant was collected for sEV extraction and purification. The cell culture supernatant was harvested and centrifuged at 300 ×g for 10 min to separate the cells. The supernatant was retained and centrifuged at 2,000 ×g for 10 min to remove dead cells. Afterward, the centrifugal force was increased to 10,000 ×g and centrifuged for 30 min to eliminate cell debris. Subsequently, the processed supernatant was subjected to ultracentrifugation at 100,000 ×g for 70 min to obtain a crude sEV pellet. The sEV pellet was resuspended in PBS and centrifuged again at 100,000 ×g for 70 min to acquire purified sEVs [20]. and stored at -80 °C for later use. The ultrastructure and size distribution of the extracted sEVs were examined using transmission electron microscopy (TEM, H-7650, Hitachi, Japan) and nanoparticle tracking analysis (NTA) using ZetaView Z-NTA (Particle Metrix) and the corresponding software (ZetaView 8.04.02).

### Flow cytometry for assessing cell transfection efficiency

When the cell confluence reached 80–90%, the cells were digested and collected. PBS (2 mL) was added and the cell pellet resuspended, followed by cell counting. Afterward, 1 × 10^^5^ cells were transferred into 1.5-mL Eppendorf tubes, washed thrice with 500 µL PBS, and resuspended in 100 µL PBS. A flow cytometer (Beckman Coulter, CA, USA) was used to measure GFP fluorescence at an excitation of 488 nm., which was compared with that of a negative control to determine the percentage of GFP-positive cells.

### Exosome uptake experiment

In the evaluation of exosome uptake efficiency, exosomes were first labeled with Dio by incubation at a temperature of 37 °C away from light for 30 min. Following the incubation, the exosomes were washed thrice with PBS and centrifuged at 100,000 × g for 70 min to remove any excess Dio stain. Thereafter, the Dio-tagged exosomes were co-incubated with 3T3-L1 cells for 3 h. Post-incubation, the cells were double-washed with PBS and fixed in 4% paraformaldehyde for 10 min on ice. Nuclei were stained with DAPI (D9542, Sigma-Aldrich; Merck KGaA), washed thrice with PBS, and imaged using a fluorescence microscope (NIKON Eclipse Ti, Japan).

### Glucose uptake assay in 3T3-L1 adipocytes

Glucose uptake by 3T3-L1 adipocytes was determined using the Glucose Uptake-Glo™ Assay, based on the detection of 2-deoxyglucose-6-phosphate (2DG6P) (Promega, Madison, WI, USA). The procedure involved overnight serum starvation of differentiated cells (day 7) in DMEM containing 4.5 g/L glucose without serum. Subsequently, the cells were preincubated for 40 min in KRPH buffer (20 mM HEPES, 5 mM KH_2_PO_4_, 1 mM MgSO_4_, 1 mM CaCl_2_, 136 mM NaCl, and 4.7 mM KCl; pH 7.4), supplemented with 2% bovine serum albumin (BSA; Bioshop, BS114), to deplete the intracellular glucose. Cells were stimulated with 1 µM insulin for 20 min and then incubated with 1 mM 2-deoxyglucose (2-DG) for another 20 min. The 2DG6P detection reagent was incubated at 25℃ for 1 h. Glucose uptake was assessed using a Tecan Spectra Fluor Plus plate reader (Thermo Fisher Scientific) and Tecan iControl 1.5 software (*N* = 6).

### Cell viability assay

A Cell Counting Kit-8 (CCK-8, HY-K0301, MCE, China) assay was employed to determine the impact of small extracellular vesicles (sEVs) on 3T3-L1 cells. Initially, 3T3-L1 cells were seeded into each well of a 96-well plate and allowed to proliferate for 24 h. Subsequently, the cells were exposed to sEVs at a concentration of 3.5 × 10^4 particles per well for either 24–48 h. After the treatment period, 100 µL of complete medium was combined with 10 µL of CCK-8 reagent in each well and incubated for 2 h. Absorbance at 450 nm was measured using a Tecan Spectra Fluor Plus plate reader and Tecan iControl 1.5 software (*N* = 3).

### Western blotting

The cells and tissues were lysed using RIPA lysis buffer (MedChemExpress, Monmouth Junction, NJ, USA, HY-K1001) containing phosphatase and protease inhibitors. To determine protein concentrations, a BCA protein assay kit (Beyotime, Shanghai, China, P0010) was used. Lysates were separated using 10% SDS gel electrophoresis and then transferred to polyvinylidene difluoride membranes (Millipore, Burlington, MA, USA, IPVH00010). The membranes were blocked with 5% BSA for 2 h at 25℃ and then incubated with primary antibodies at 4 °C overnight. The primary antibodies used in this study are listed in Table 1. Subsequently, the membrane was incubated with a secondary antibody for 1.5 h at room temperature. Protein bands were detected using an electrochemiluminescence assay reagent (P0018S; Beyotime) (*N* = 6 for in vivo experiments, *N* = 3 for in vitro experiments).

**Table 1:** Corresponding antibodies for Western blotting

Antibody	Dilution	Company	Cat #
Anti-Akt	1:1000’	Affinity Biosciences	AF0836
Anti-p-Akt	1:10000	Affinity Biosciences	AF0016
Anti-ACTB	1:1000’	Affinity Biosciences	AF7018
Anti-AMPK	1:1000’	Affinity Biosciences	AF6423
Anti-p-AMPK	1:1000’	Affinity Biosciences	AF3423
Anti-GLUT4	1:1000’	Affinity Biosciences	AF5386
Anti-Apelin	1:2500	Abcam	ab125213
Anti-CD63	1:2000	Abcam	ab134045
Anti-CD81	1:2000’	Abcam	ab79559
Anti-TSG101	1:2000’	Abcam	ab125011
Anti-Calnexin	1:1000’	Abcam	ab22595
Secondary Antibody	1:10000’	Affinity Biosciences	S0001
Secondary Antibody	1:10000’	Affinity Biosciences	S0002

### Quantitative reverse transcriptase-PCR (qRT-PCR)

Total RNA was extracted using TRIzol reagent (Tiangen Biotech, China, DP424) and then reverse-transcribed to complementary DNA using the Evo M-MLV RT Reaction Mix (AG, AG11728). Quantification was performed using the ChamQ Universal SYBR qPCR Master Mix (Novozymes, Q711-03). Each sample was analyzed in triplicate using the CFX Connect Real-Time PCR Detection System (Bio-Rad). The relative expression levels of RNA were calculated using the 2^−ΔΔCt^ method. The primers used in this study are listed in Table 2 (*N* = 6).

**Table 2:** Primer sequences used for qRT-PCR

Gene	Primer sequences	Fragment (bp)
*apelin-F*	5′-GTCTCCTCCATAGATTGGTCTGC − 3′	149
*apelin-R*	5′-GGAATCATCCAAACTACAGCCAG-3′	
*β-actin-F*	5′-CATGTACGTTGCTATCCAGGC − 3′	250
β-actin-R	5′-CTCCTTAATGTCACGCACGAT − 3′	

### T2DM mouse model

C57BL/6J mice were provided by the SPF Biotechnology Company (Beijing, China). Animal procedures followed the institutional guidelines, and the research design was approved by the Experimental Animal Ethics Committee of Beijing GENE LINE Biotechnology Co. Ltd. (permit Number JLSW-20230623-01).

A T2DM mouse model was established using a high-fat diet combined with streptozotocin (STZ). To induce diabetes, a single intraperitoneal injection of STZ (60 mg/kg, dissolved in 0.1 mol/L citrate buffer, pH 4.5) was administered to the experimental group of mice. The control group was administered an equivalent volume of saline solution. Blood glucose levels in the tail veins of the mice were measured every three days throughout the experiment. Blood was collected from the distal segment of the tail vein, ensuring a minimum volume of not less than 2 µL for each measurement to ensure the accuracy of glucose readings. Mice with blood glucose levels < 16 mM were excluded from the experimental group. At the end of the experiment, mice were euthanized using a lethal dose of ether. The pancreas of each mouse was removed via a midline abdominal incision and fixed in 10% formalin saline for 24 h. Fixed tissues were processed in paraffin blocks for subsequent studies.

### Hematoxylin and eosin (H&E) staining

H&E staining involved the following steps: tissue sections were immersed overnight in 4% paraformaldehyde solution at 4 °C, followed by dehydration and embedding in paraffin. The embedded sections were dewaxed and rehydrated. To perform H&E staining, we used the Solarbio H&E Staining Kit (cat # G1120-100) following the manufacturer-recommended protocol. H&E staining was performed using a commercial kit according to the manufacturer’s instructions (Solarbio, G1120-100). The methods of security detection with HE are reported in a previous study [21]. This procedure was repeated three times for each experimental group (*N* = 3).

### Immunofluorescence

Immunofluorescence was used to assess the effects of lentiviral transfection on the cells. First, cells were washed with cold PBS and fixed with 4% paraformaldehyde for 15 min. Subsequently, the cells were blocked with 5% serum at 37 °C for 60 min before incubation with anti-Apelin antibody (1:2500 dilution; Abcam, Cat# ab125213) at 4 °C overnight. Following incubation, the cells were subjected to three PBS washes and then exposed to a secondary antibody at a 1:100 dilution (1:500, Cat #4412; CST) at 37 °C for 2 h. For nuclear staining, 2-(4-amidinophenyl)-1 H-indole-6-carboximidamide (DAPI; D9542, Sigma-Aldrich; Merck KGaA) was applied for 2 min at 37 °C. After another series of three PBS washes, the cells were incubated overnight at 4 °C. Sections were deparaffinized and rehydrated, and antigens were retrieved using 10 mM Tris/EDTA (pH 9.0). The sections were permeabilized and blocked in PBS containing 1% BSA and 5% goat serum. Primary antibodies were incubated overnight at 4 °C, followed by incubation with fluorophore-conjugated secondary antibodies at 37 °C for 1 h. Pancreatic sections were stained with an anti-insulin antibody (1:800; GB15336-100, Servicebio) and anti-ki67 (1:200; GB121141-100, Servicebio), followed by counterstaining with tyramine and amplification using fluorophores 488 and CY3 and DAPI to determine the proliferation of β cells. Insulin-positive (insulin+) cells showing nuclear colocalized staining for DAPI + and ki67 + were considered proliferating β cells. Sections were visualized using a fluorescence microscope (NIKON Eclipse Ti, NIKON, Tokyo, Japan) (*N* = 6 for in vivo experiments, *N* = 3 for in vitro experiments).

### Oral glucose tolerance test (OGTT) and intraperitoneal insulin tolerance test (IPITT)

Mice were subjected to OGTT and IPITT five weeks post-treatment with PBS, apelin-13, MSC-sEVs, or AP-MSC-sEVs. For the OGTT, mice were fasted for 16 h, followed by the oral administration of D-glucose (2 g/kg). Blood glucose levels were measured at 0, 15, 30, 60, 90, and 120 min after oral glucose administration (2.0 g/kg), using an Accu-Chek Performa Blood Glucose Meter. For the IPITT, mice were fasted for 6 h and then received an intraperitoneal injection of insulin (1 U/kg). Blood glucose levels were measured at 0, 15, 30, 60, 90, and 120 min after the insulin injection. The methodology for calculating the area under the curve (AUC) is based on those in previous studies [20, 22] ( *N* = 3 ).

### Cellular inflammatory cytokines assessment

Cytokines and chemokines in the mouse serum were quantified using a cytometric bead array (CBA) in the LEGENDplex Mouse Inflammation Panel (BioLegend, 740446) following the manufacturer’s instructions. The data collected from this assay were analyzed using the LEGENDplex™ software (BioLegend).

### Statistical analysis

Statistical analyses were performed using SPSS version 17.0. Data are presented as mean ± standard deviation. Student’s t-tests were employed for comparing the data of two groups, while a one-way ANOVA was used for comparisons among three or more groups. *P* < 0.05 was considered statistically significant.

## Results

### Overexpression of apelin in WJ-MSCs

**Fig. 1:**
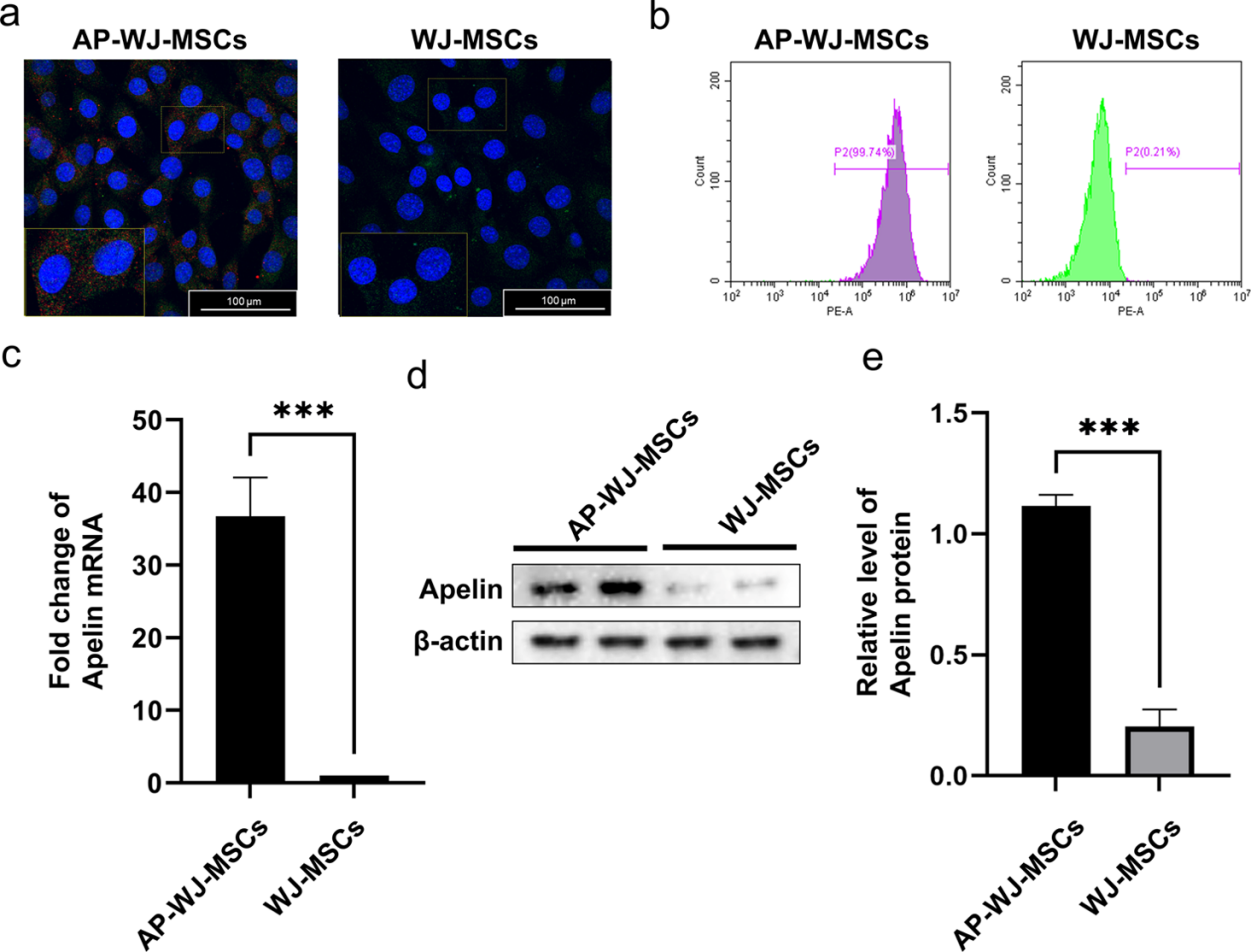
Overexpression of apelin in WJ-MSCs and analysis of transduction efficiency. (**a**) Immunofluorescence images showing the intracellular GFP fluorescence in AP-WJ-MSCs (Blue: nuclear DAPI, Red: Apelin, Green: GFP). (**b**) Quantitative flow cytometric analysis confirming the efficiency of lentiviral transduction in WJ-MSCs. (**c**) qRT-PCR results indicating a 36.74-fold increase in Apelin gene expression in Ap-MSC-sEVs compared to that in MSC-sEVs (***P < 0.001). (**d**–**e**) Western blot images depicting the protein expression levels of apelin in AP-MSC-sEVs (***P < 0.001). *Abbreviations*: sEVs, small extracellular vesicles; AP-MSC-sEVs, apelin-MSC-sEVs, engineered sEVs loaded with overexpressed apelin; Wharton’s jelly-derived mesenchymal stem cells (WJ-MSCs)

Lentiviral particles containing apelin were prepared using HEK 293T cells. After concentration and titration, these lentiviral particles were used to transduce WJ-MSCs, and intracellular GFP fluorescence was observed via fluorescence microscopy **(**Fig. 1a**)**. The efficiency of this transduction, which was observed to be over 99% under a fluorescence microscope, was further confirmed using quantitative flow cytometric analysis **(**Fig. 1b**)**. Additionally, the expression of the apelin gene in WJ-MSCs after lentiviral transduction was evaluated using qRT-PCR. Our results indicated the successful expression of apelin in the transduced cells. Compared to that in the control WJ-MSCs, there was a 36.74-fold increase in apelin expression in Ap-WJ-MSCs (*P* < 0.05) **(**Fig. 1c**)**. Finally, the results of western blotting revealed significant expression of apelin in transduced WJ-MSCs (*P* < 0.05) **(**Fig. 1d, e**)**.

### sEV extraction and characterization

**Fig. 2:**
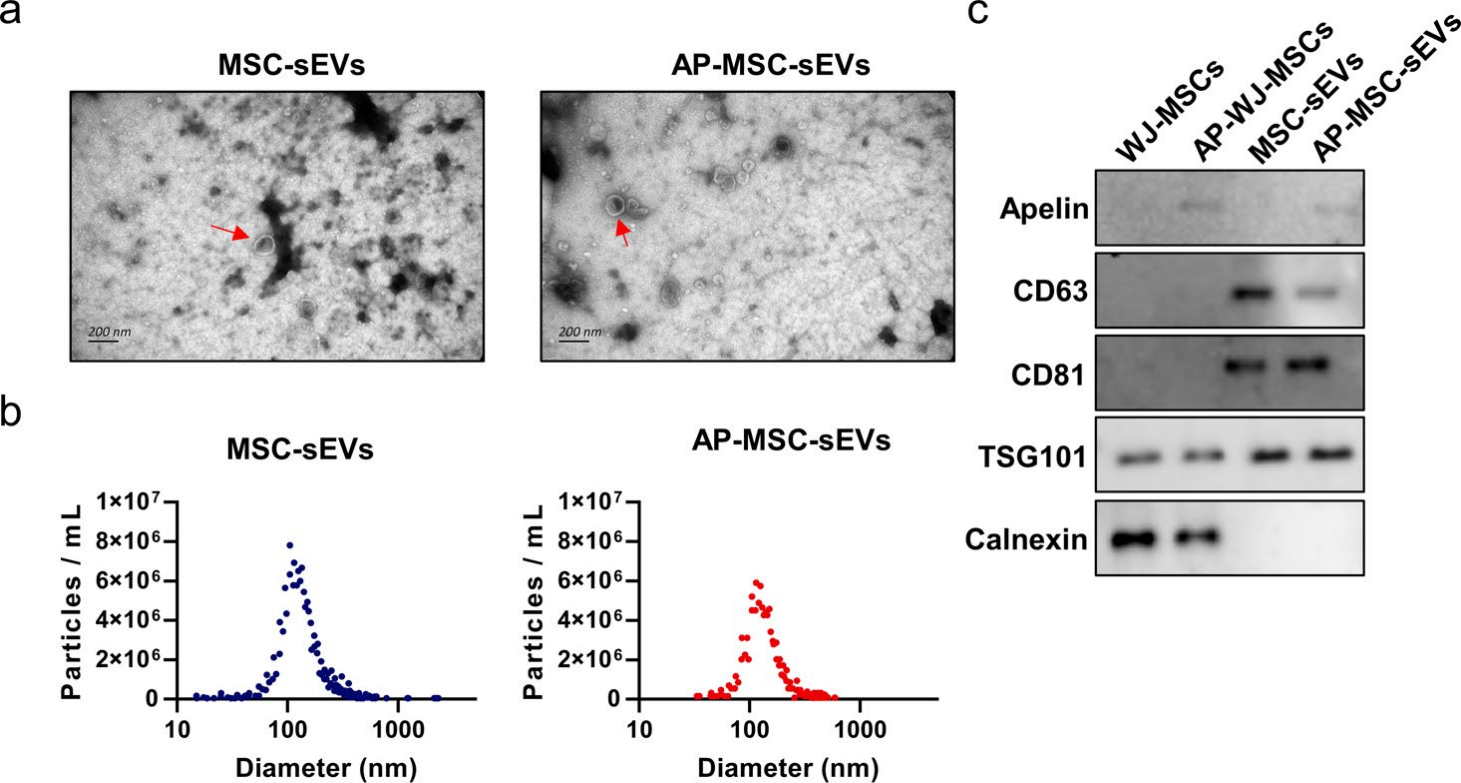
Characterization of sEVs derived from WJ-MSCs and apelin-modified WJ-MSCs. (**a**) TEM image showing the typical cup-shaped morphology of the extracted sEVs. (**b**) Nanoparticle tracking analysis (NTA) displaying an average sEV diameter of 100–120 nm. (**c**) Western blot results indicating positive expression of sEV markers CD63, CD81, and TSG101 and negative expression of the non-sEVs marker calnexin blot as well as the presence of apelin protein in Ap-MSC-sEVs. *Abbreviations*: sEVs, small extracellular vesicles; AP-MSC-sEVs, apelin-MSC-sEVs, engineered sEVs loaded with overexpressed apelin; Wharton’s jelly-derived mesenchymal stem cells (WJ-MSCs); TEM, transmission electron microscopy

sEVs extracted from the cell culture supernatant were examined under a transmission electron microscope (TEM), which revealed a typical cup-shaped morphology **(**Fig. 2a**)**. NTA indicated that the sEVs had an average diameter of 100–120 nm **(**Fig. 2b**)**. These findings confirmed the successful extraction of sEVs. Both WJ-MSC-sEVs (MSC-sEVs) and apelin-WJ-MSC-sEVs (AP-MSC-sEVs) displayed membranous vesicles under a TEM, while western blotting demonstrated positive expression of the sEV markers CD63 and CD81 as well as TSG101 on the surface of sEVs. In contrast, the non-sEV marker calnexin showed negative expression. Furthermore, apelin protein expression was observed in the AP-MSC-sEVs **(**Fig. 2c**)**.

### Impact of sEVs on glucose uptake in IR cell model

**Fig. 3:**
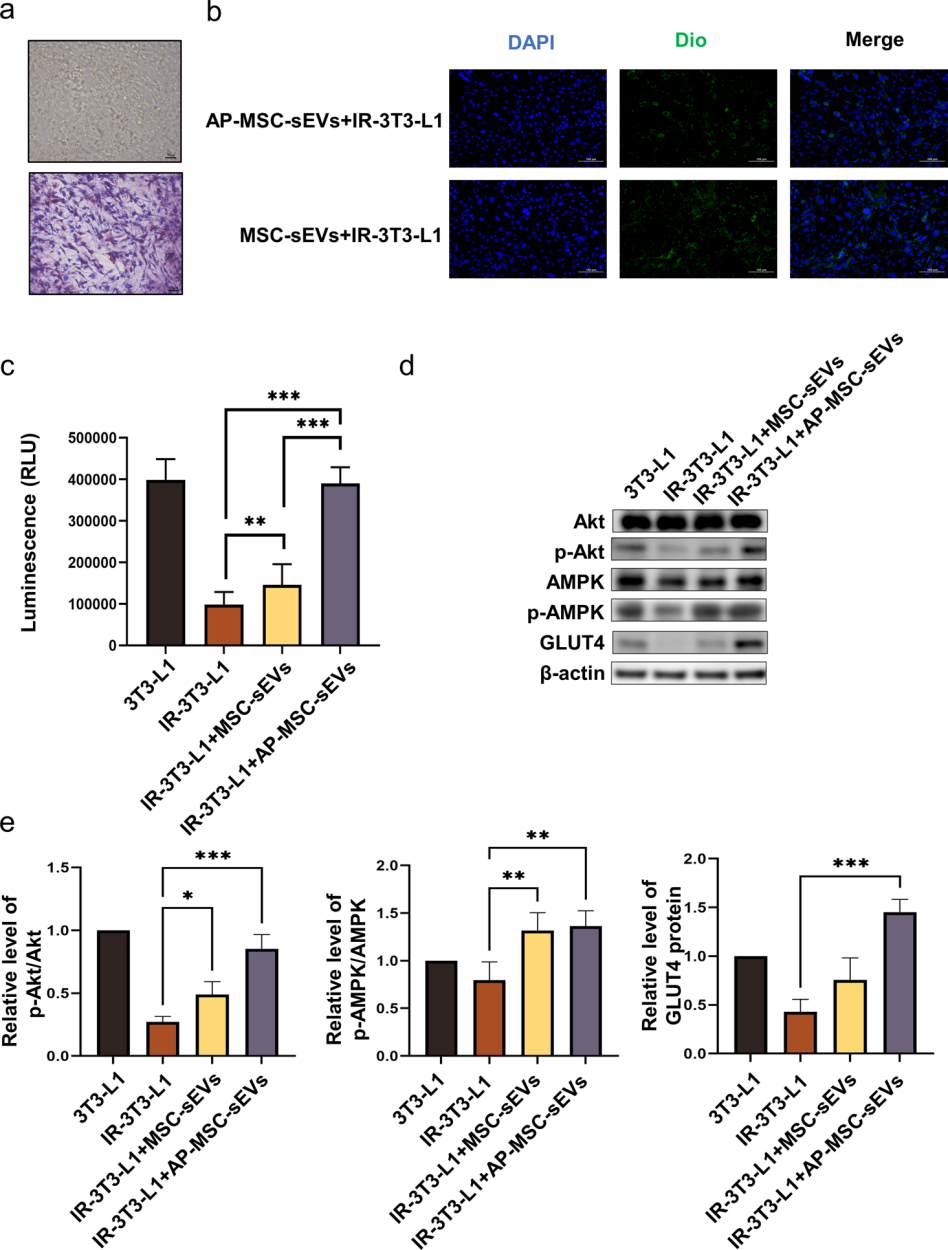
Effects of WJ-MSC- and apelin-modified WJ-MSC-sEVs on IR in 3T3-L1 cells. (**a**) Oil Red “O” staining of 3T3-L1 cells showing significant lipid droplet formation. (**b**) Confocal microscopic images depicting the uptake of both MSC-sEVs and AP-MSC-sEVs by 3T3-L1 cells. (**c**) Comparison of glucose uptake in IR-3T3-L1 cells treated with MSC-sEVs, AP-MSC-sEVs, and controls, showing enhanced glucose uptake, especially with AP-MSC-sEVs (*P* < 0.05). (**d**–**e**) Western blot images showing the impact of sEVs treatment on p-Akt/total Akt and p-AMPK/total AMPK expression levels and enhancement of membrane GLUT4 protein levels in insulin-resistant cells (*P < 0.05, **P < 0.01, ***P < 0.001). *Abbreviations*: sEVs, small extracellular vesicles; AP-MSC-sEVs, apelin-MSC-sEVs, engineered sEVs loaded with overexpressed apelin; Wharton’s jelly-derived mesenchymal stem cells (WJ-MSCs)

An IR cell model was constructed using 3T3-L1 cells in which significant lipid droplet formation was observed. Under Oil Red “O” staining, adipocytes were distinctly marked **(**Fig. 3a**)**. We co-cultured MSC-sEVs and AP-MSC-sEVs with 3T3-L1 cells for 12 h (1 × 10^6^ particles per well of a 6-well plate), observed them using confocal microscopy, and found that both kinds of sEVs were taken up by the 3T3-L1 cells **(**Fig. 3b**)**. Through CCK-8 assay, we demonstrated that the two sEVs did not affect cell proliferation (Figure S1). To investigate the effects of MSC-sEVs and AP-MSC-sEVs on insulin sensitivity and glucose uptake in IR-treated 3T3-L1 cells (IR-3T3-L1), an insulin-resistant cell line, both types of sEVs were applied to these cells, with PBS-treated 3T3-L1 cells as the negative control and IR-3T3-L1 cells as the positive control. The results demonstrated that glucose uptake in IR-3T3-L1 cells was significantly reduced compared to that in the control group (*P* < 0.05). Both MSC-sEVs and AP-MSC-sEVs significantly improved glucose uptake, with AP-MSC-sEVs exhibiting a more pronounced effect (*P* < 0.05) **(**Fig. 3c**)**. We observed a reduction in phosphorylated protein kinase B (p-Akt)/total Akt expression levels in IR-3T3-L1 cells, which was ameliorated by the MSC-sEV treatment and even more so by the AP-MSC-sEV treatment (Fig. 3d, e). A similar pattern was observed for protein kinase (p-AMPK)/total AMPK expression levels (Fig. 3d, e). GLUT4 levels were reduced in cells in the IR-3T3-L1 group compared to those in the control group, but GLUT4 levels were increased after both sEV treatments, especially in the AP-WJ-sEVs group (*P* < 0.05) **(**Fig. 3d, e**)**.

### Validation of the therapeutic efficacy of sEVs injection in T2DM mice

**Fig. 4:**
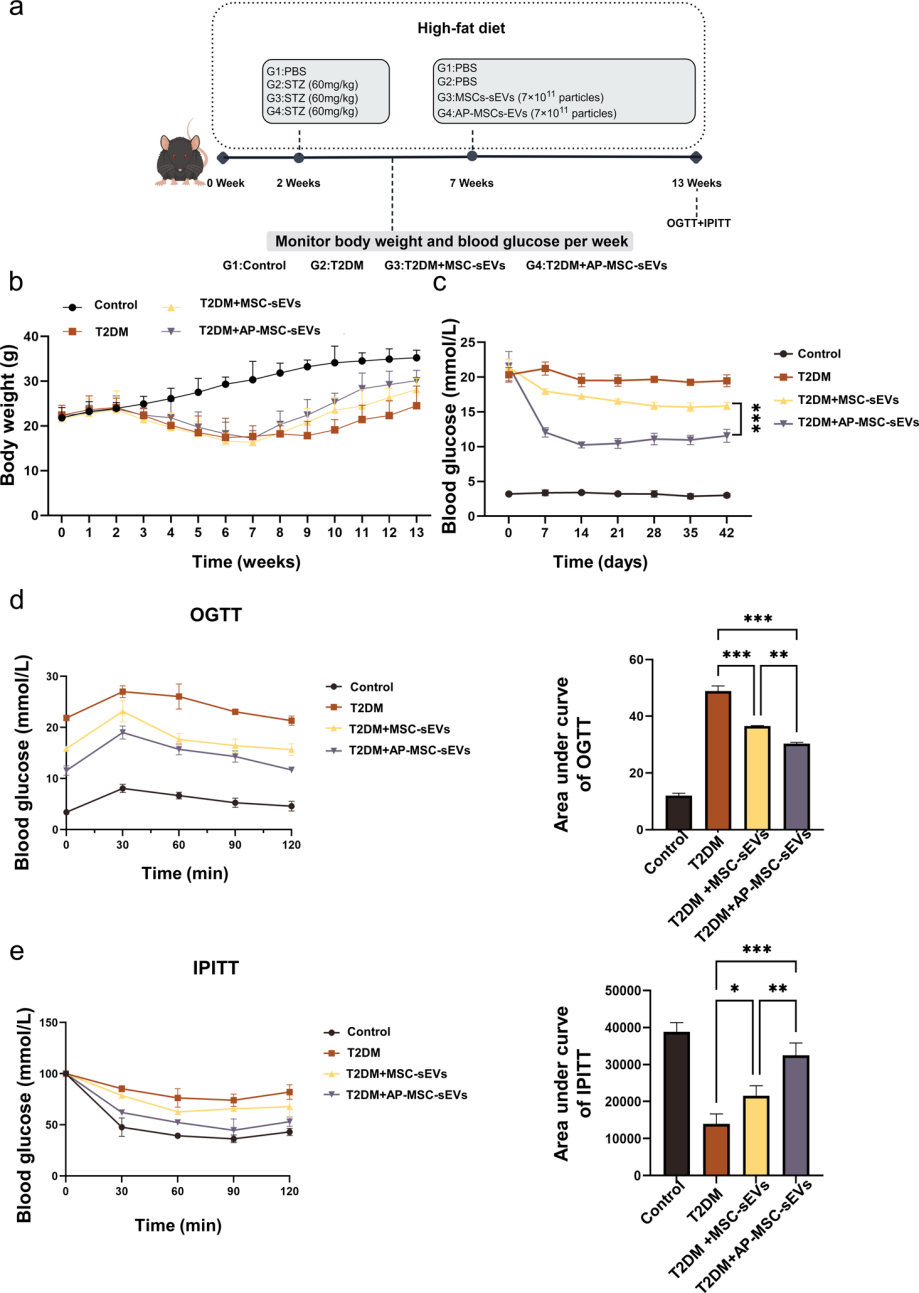
Therapeutic efficacy of sEV treatment in T2DM mouse model. (**a**) Timeline of mouse treatment and research. (**b**) Changes in body weights of mice over time. (**c**) Blood glucose levels post-intravenous injection of MSC-sEVs and Ap-MSC-sEVs, or PBS as control; a significant reduction was observed in glucose levels, particularly with Ap-MSC-sEVs. (**d**–**e**) OGTT and IPITT results indicating improved glucose homeostasis in sEV-treated mice, with AP-MSC-sEVs showing increased efficacy compared to that of MSC-sEVs (*P < 0.05, **P < 0.01, ***P < 0.001). *Abbreviations*: sEVs, small extracellular vesicles; AP-MSC-sEVs, apelin-MSC-sEVs, engineered sEVs loaded with overexpressed apelin; Wharton’s jelly-derived mesenchymal stem cells (WJ-MSCs)

To investigate the therapeutic effects of MSC-sEVs and Ap-MSC-sEVs in vivo, a T2DM mouse model was established. Tail vein injections of MSC-sEVs and Ap-MSC-sEVs were administered to the experimental groups (7 × 10^11^ particles per mouse), while the control group (C57BL/6J mice) received PBS **(**Fig. 4a**)**. Safety evaluation in mice was conducted by HE staining of various internal organs on day 3 after tail vein injection to evaluate biocompatibility (Figure S2). Under the high-fat diet, the weight of control group increased consistently, whereas the T2DM group experienced a rapid decrease in weight following STZ injection. Following sEV injections, both groups demonstrated more rapid weight recovery than the T2DM group. Notably, the weight recovery rate in the AP-MSC-sEVs group surpassed that observed with the MSC-sEV injection.T2DM mice treated with sEVs showed a significant reduction in blood glucose levels, although the Ap-MSC-sEVs treatment exhibited a more pronounced effect in terms of reducing blood sugar levels, than the MSC-sEVs treatment, and maintaining better glucose stability post-injection **(**Fig. 4c**)**. Furthermore, the OGTT and IPITT were conducted to assess the impact of the treatments on glucose homeostasis. At the end of the intervention period, T2DM mice showed significantly increased fasting blood glucose levels and reduced glucose tolerance, reflected by the AUC during both the OGTT and IPITT. As expected, treatment with both MSC-sEVs and AP-MSC-sEVs improved glucose homeostasis in mice (Fig. 4d, e).

### Restoration of pancreatic β-cell function

**Fig. 5:**
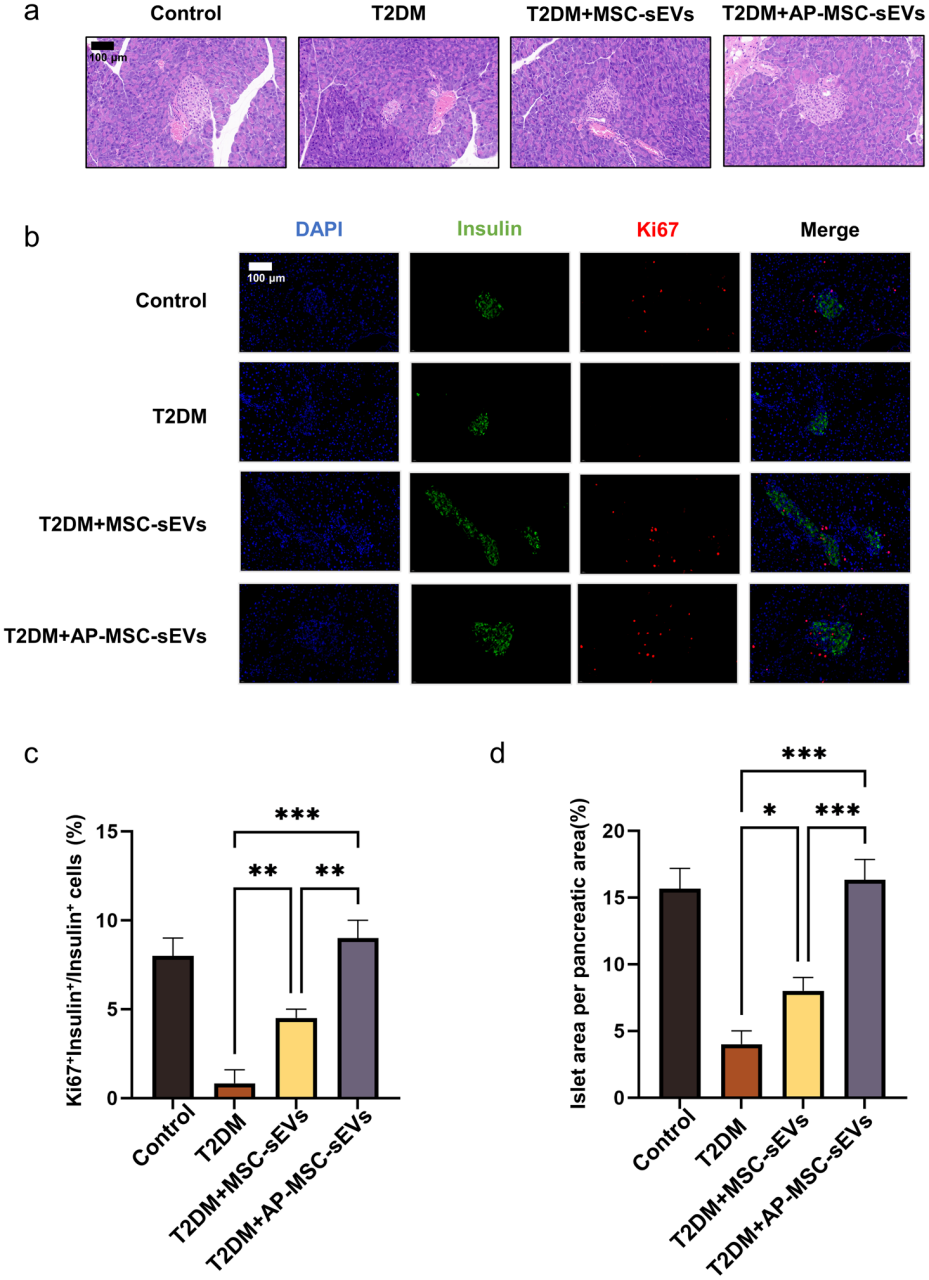
Effects of MSC-sEVs and Ap-MSC-sEVs on pancreatic β-cell function in T2DM mice. (**a**) Increased β-cell area in pancreatic tissues (*P* < 0.05). (**b**) Presence of more insulin-positive and Ki-67-positive β-cells, indicating cell replication in Ap-MSC-sEV treatment (*P* < 0.05). (**c**) Increased number of Ki67^+^Insulin^+^/Insulin^+^ cells in the Ap-MSC-sEV-treated group (*P* < 0.05). (**d**) Comparison of islet area and Ki-67 positive cell count between treatments (*P < 0.05, **P < 0.01, ***P < 0.001). *Abbreviations*: sEVs, small extracellular vesicles; AP-MSC-sEVs, apelin-MSC-sEVs, engineered sEVs loaded with overexpressed apelin; Wharton’s jelly-derived mesenchymal stem cells (WJ-MSCs)

Examination of the pancreatic tissue of mice treated with MSC-sEVs and Ap-MSC-sEVs revealed an increase in pancreatic β-cell area (*P* < 0.05) (Fig. 5a). To identify the cause of the increase in β-cell area, pancreatic sections were subjected to immunofluorescence staining to identify the cells stained with Ki-67, an indicator of actively replicating cells. Compared with the C57BL/6J mice, the T2DM mice injected with PBS and those injected with MSC-sEVs and Ap-MSC-sEVs exhibited a significant increase in the number of insulin-positive and Ki-67-positive cells within the islets, which suggested that the increased replication of β-cells likely drove the enlargement of the β-cell area (Fig. 5b) (*P* < 0.05). Furthermore, the increase in the number of Ki67^+^Insulin^+^/Insulin^+^ cells was more pronounced in the Ap-MSC-sEV-treated group than in the MSC-sEV-treated group (*P* < 0.05) (Fig. 5c). Finally, the Ap-MSC-sEV-treated group had a greater number of Ki-67 positive cells (*P* < 0.05) (Fig. 5d), although the islet area per pancreatic area in the mice injected with MSC-sEVs was larger than that in the Ap-MSC-sEV-injected group.

### In vivo adipose tissue analysis

**Fig. 6:**
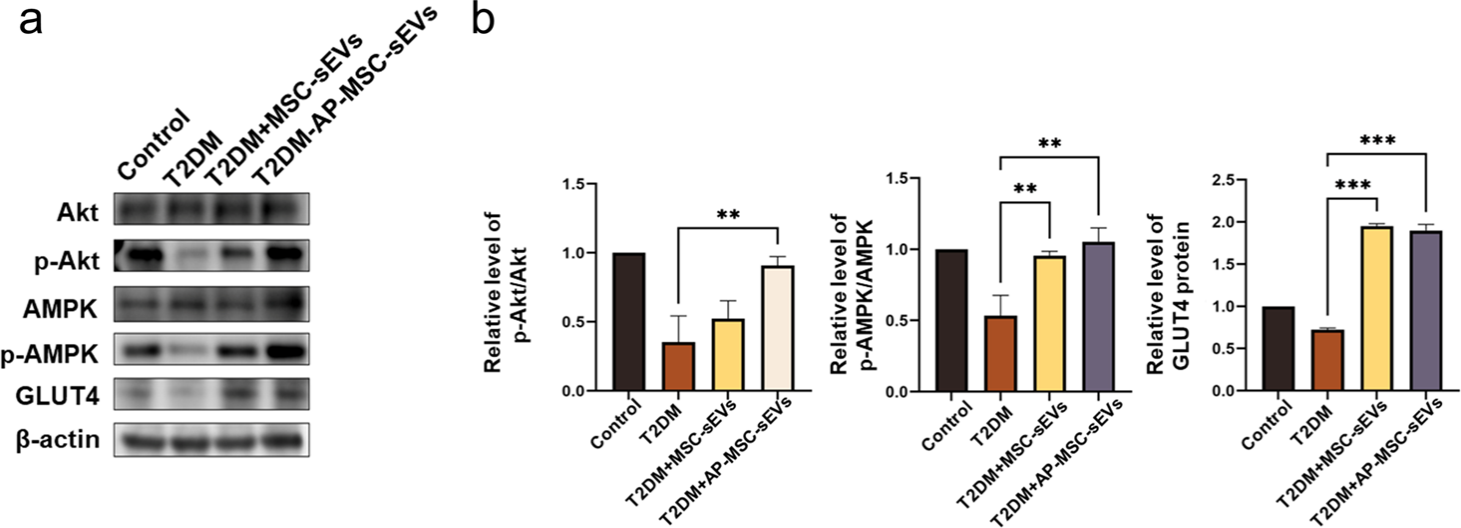
Analysis of adipose tissue in T2DM model mice treated with MSC-sEVs and AP-MSC-sEVs. (**a**–**b**) SDS-PAGE results showing the effects of sEV treatments on AMPK and Akt phosphorylation. MSC-sEV and AP-MSC-sEV treatments enhanced AMPK phosphorylation and the p-Akt/Akt ratio, indicating improved insulin sensitivity (*P* < 0.05); more pronounced phosphorylation effect was observed with AP-MSC-sEVs. GLUT4 protein expression also significantly increased following sEV treatments (*P < 0.05, **P < 0.01, ***P < 0.001). *Abbreviations*: sEVs, small extracellular vesicles; AP-MSC-sEVs, apelin-MSC-sEVs, engineered sEVs loaded with overexpressed apelin; Wharton’s jelly-derived mesenchymal stem cells (WJ-MSCs)

The protein levels of AMPK, p-AMPK, Akt, p-Akt, and GLUT4 in the adipose tissue were assessed using western blotting. In T2DM mice, AMPK phosphorylation levels were reduced, which was significantly compensated for by both MSC-sEV and Ap-MSC-sEV treatments. Furthermore, Akt phosphorylation was examined to evaluate the effect of these sEVs on IR. Compared to the control or PBS-injected T2DM mice, both MSC-sEV- and Ap-MSC-sEV-treated mice showed enhanced insulin sensitivity, driven by an increase in the p-Akt/Akt ratio (*P* < 0.05). Moreover, the Ap-MSC-sEV-treated mice presented significantly higher levels of phosphorylated Akt than the MSC-sEV-treated T2DM mice **(**Fig. 6a, b**)**. GLUT4 protein levels were also significantly elevated (*P* < 0.05) after treatment with MSC-sEVs and Ap-MSC-sEVs.

### Inflammatory cytokine analysis

**Fig. 7:**
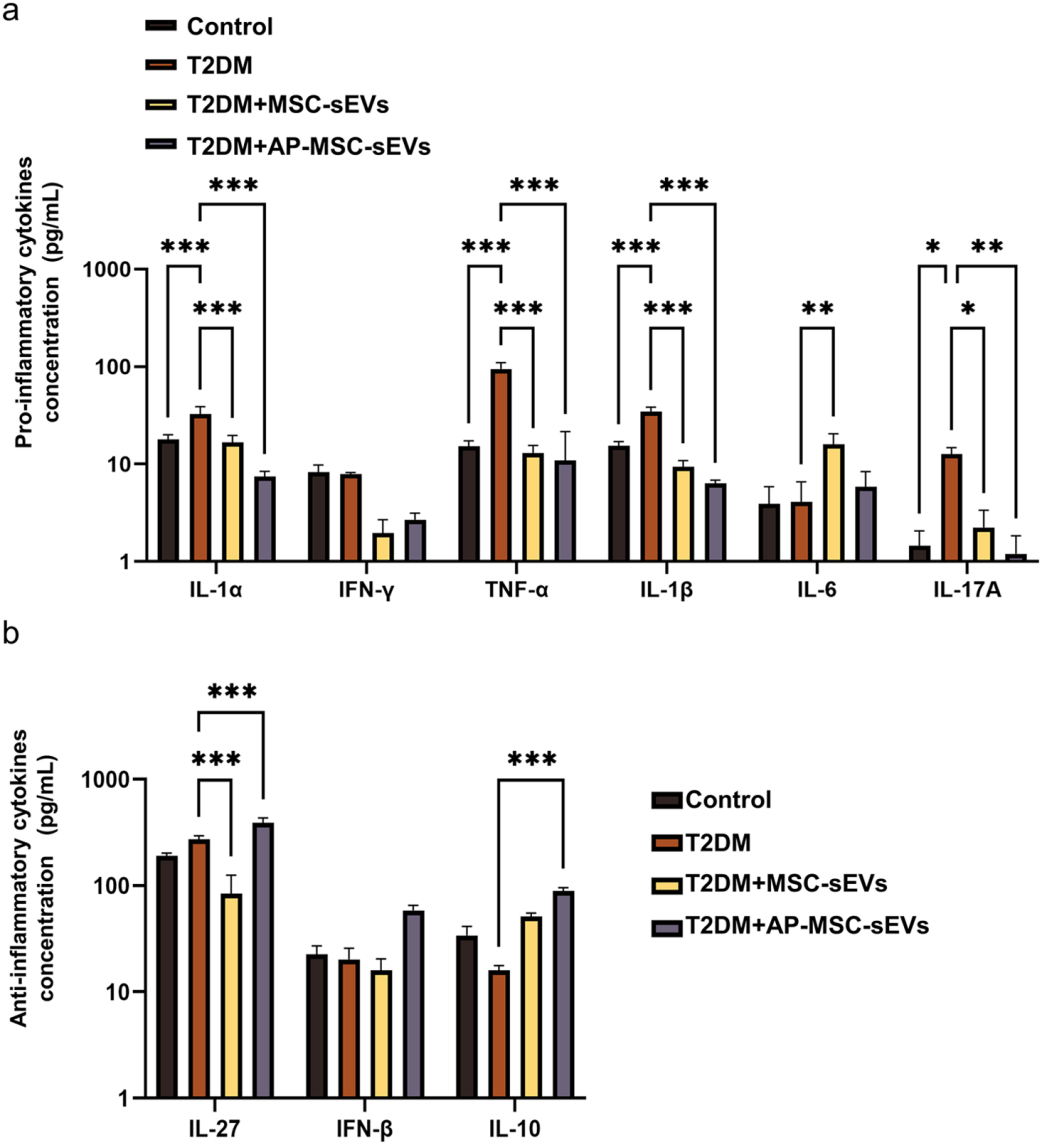
Inflammatory cytokine levels in T2DM model mice treated with MSC-sEVs and AP-MSC-sEVs. (**a**) Reduction in pro-inflammatory cytokine levels with both treatments, although more significantly with AP-MSC-sEVs treatment (*P* < 0.05), except for IL-6 in the MSC-sEVs group. (**b**) Serum levels of the anti-inflammatory factor IL-27 decreased slightly, whereas those of IL-10 increased in the T2DM + PBS group; there were significant changes in the levels of the factors under both sEV treatments, with a notable increase in IFN-β levels only in the T2DM + AP-MSC-sEVs group (*P* < 0.05; *P < 0.05, **P < 0.01, ***P < 0.001). *Abbreviations*: sEVs, small extracellular vesicles; AP-MSC-sEVs, apelin-MSC-sEVs, engineered sEVs loaded with overexpressed apelin; Wharton’s jelly-derived mesenchymal stem cells (WJ-MSCs)

CBA revealed that proinflammatory cytokine levels were generally higher in the T2DM + PBS group than in the control group. Notably, the levels of most pro-inflammatory cytokines, except for IL-6, were further reduced in the T2DM + MSC-sEVs group (*P* < 0.05), which suggests that the MSC-sEVs treatment has a significant inhibitory effect on specific cytokines. In the T2DM + Ap-MSC-sEVs group, the levels of all pro-inflammatory cytokines were significantly reduced, suggesting a potent anti-inflammatory effect of Ap-MSC-sEVs (*P* < 0.05) **(**Fig. 7a**)**. Further analysis of the serum levels of the anti-inflammatory factors IL-27, IFN-β, and IL-10 showed that in the T2DM + PBS group, there was a slight decrease in the levels of IL-27 and a significant increase in IL-10 compared to those in the control group (*P* < 0.05). In the T2DM + MSC-sEVs group, IL-27 and IL-10 levels significantly decreased and increased, respectively. In contrast, IL-27 levels were significantly higher in the T2DM + Ap-MSC-sEVs group (*P* < 0.05). Meanwhile, IFN-β levels showed a significant increase only in the T2DM + Ap-MSC-sEVs group **(**Fig. 7b**)**.

## Discussion

Recent research has highlighted the significant role of continuous apelin infusion in improving serum insulin levels and reducing hyperglycemia in rats with T2DM induced by a high-fat diet and STZ [23]. Previous studies have demonstrated that continuous infusion of apelin-overexpressing WJ-MSCs can elevate insulin and C-peptide levels in the plasma of T2DM rats, indicating a potential therapeutic effect on the metabolic dysfunction characterizing this condition [12]. The strategy of repairing islet beta cells through various reprogramming strategies is an important research direction [24]. Building upon such findings, the present study further elucidated the therapeutic potential of apelin not only in modulating serum insulin levels but also in directly enhancing pancreatic β-cell proliferation.

The present study expands the understanding that MSCs, particularly those overexpressing apelin, have a profound impact on diabetes and obesity management [25]. We observed that MSC-derived sEVs, specifically those from WJ-MSCs overexpressing apelin, effectively enhance insulin sensitivity, increase glucose uptake in peripheral tissues, and inhibit hyperglycemia and β-cell apoptosis. The application of sEVs presents a novel mechanism for extending the therapeutic benefits of mesenchymal stem cells in T2DM treatment.

We confirmed that treatment with Ap-MSC-sEVs influenced critical molecular pathways, including those related to the phosphorylation of AMPK and Akt. The findings provide insights into the molecular basis of the therapeutic effects of Ap-MSC-sEVs in T2DM. In previous studies, MSCs have been shown to alleviate the muscle atrophy caused by diabetes and obesity through sEV-mediated enhancement of AMPK/ULK1-driven autophagy [25], thereby extending the therapeutic duration of stem cells [26]. Furthermore, sEVs from MSCs have been shown to enhance insulin sensitivity in T2DM rats, increase glucose uptake and metabolism in peripheral tissues, and inhibit hyperglycemia and β-cell apoptosis induced by STZ [27]. Our study further demonstrated that WJ-MSCs overexpressing apelin exhibited enhanced effects, including prolongation of the action of apelin and enhancement of the efficacy of MSC-sEVs. Moreover, previous studies suggested that apelin affects the phosphorylation of AMPK and Akt [28]. Our research confirmed that Ap-MSC-sEVs can influence the same processes. We also found that Ap-MSC-sEVs affect the translocation mechanisms of GLUT4 in adipocytes and glucose metabolism and that sEVs modulate fatty inflammation by regulating Akt [29, 30].

Metabolic inflammation and changes in adipokine dynamics are closely linked to local and systemic inflammation and IR, which are key factors involved in metabolic syndrome [31, 32]. Hypoglycemic drugs such as dagaglizin promote islet beta cell regeneration, which may be related to the increase of L-tryptophan and GLP-1 levels [33].The modulation of inflammatory cytokines, including IL-6, IL-1β, and TNF-α, by Ap-MSC-sEVs suggests the potential role of the sEVs in managing obesity-induced inflammation and metabolic syndrome. Our study showed that Ap-MSC-sEVs not only improved glucose and lipid metabolism but also reduced the damage caused by oxidative stress and inflammation possibly through the PI3K/Akt pathway. Additionally, the changes observed in IL-27, IFN-γ, and IL-17 A levels suggest that Ap-MSC-sEVs might play a role in adipose tissue inflammation and remodeling. Specifically, obesity-induced adipose tissue (AT) upregulates pro-inflammatory interleukins (IL), leading to chronic low-grade inflammation and adipocyte dysfunction [34]. IL-1α and IL-1β exacerbate IR in obesity by impairing adipocyte function and promoting inflammation [35]. Therefore, the improvement in inflammatory factors such as IL-6, IL-1β, and TNF-α by Ap-MSC-sEVs may be driven by the activation of the PI3K/Akt pathway, thereby improving glucose and lipid metabolism and reducing the damage caused by oxidative stress and inflammation [36]. Furthermore, IL-27 plays a significant role in coordinating metabolic processes and influencing metabolic morbidity [37], while IFN-γ is affected by adipose tissue inflammation, remodeling, and lipid metabolism. IL-17 A and IFN-γ are considered pro-inflammatory factors in metabolic disorders [38–40]. IL-17 A accelerates inflammation in obese mice via the TBK1/IKBKE pathway [41], whereas IFN-γ promotes preadipocyte apoptosis and inhibits adipogenesis, with IL-17 A promoting adipogenesis in vitro [42]. Although IL-17 A plays a secondary role in the inflammatory environment, it substantially contributes to the proliferation of inflammation in obese adipose tissues [43].

In the present study, the engineered sEVs significantly impacted the diseases related to cell proliferation and metabolism by modulating the PI3K/Akt/mTOR pathway and TNF-α levels, which are directly linked to the damage associated with these pathways, as mentioned in prior studies [44]. Our research further confirmed that Ap-MSC-sEVs can modulate the NF-κB pathway by affecting TNF-α, thereby mitigating inflammation and improving IR [45]. This discovery aligns with the observation that intermittent energy restriction may effectively reduce obesity-related inflammation (including IL-1β and IFN-γ) and enhance insulin sensitivity compared to continuous energy restriction. Our results also suggest that Ap-MSC-sEVs may positively influence the elevated serum levels of IL-1β, IFN-β, and IFN-γ caused by high-fat diets, which may be related to the reduced secretion of IFN-β and IL-10 induced by IL-4 in high-fat-fed mice [46]. Our findings not only emphasize the potential of Ap-MSC-sEVs in regulating inflammation and improving IR but also reveal their role in modulating the key molecular pathways associated with obesity, diabetes, and metabolic syndrome, offering new directions for developing novel therapeutic strategies in future.

Although the present study demonstrated that engineered sEVs derived from WJ-MSCs loaded with apelin exhibit T2DM treatment potential, several limitations merit attention. The use of a mouse model, although insightful, may not fully encapsulate the complexity of T2DM progression in humans, necessitating cautious interpretation of translatability. Our understanding of the long-term therapeutic effects remains unsatisfactory, with persisting questions about sustainability and potential immune responses to chronic treatment. Additionally, the mechanisms through which Ap-MSC-sEVs exert their effects require further elucidation to enhance treatment specificity and efficacy. Addressing such research gaps would facilitate advancements in the clinical application of Ap-MSC-sEVs in the management of T2DM and its metabolic complications.

## Conclusions

In conclusion, our study provides proof-of-concept for the utility of apelin-overexpressing WJ-MSCs and their derived sEVs in T2DM treatment. These findings not only contribute to a deeper understanding of the molecular mechanisms involved in T2DM but also pave the way for developing novel approaches for the treatment of this increasingly prevalent disease.

### Electronic supplementary material

Below is the link to the electronic supplementary material.

Supplementary Material 1

## Data Availability

No datasets were generated or analysed during the current study.

## Abbreviations

T2DM: type 2 diabetes mellitus
WJ-MSCs: Wharton’s jelly-derived mesenchymal stem cells
sEVs: Small extracellular vesicles
apelin-MSC-sEVs: Engineered sEVs loaded with overexpressed apelin
IR: Insulin resistance
eGFP: Enhanced green fluorescent protein
NTA: Nanoparticle tracking analysis
TEM: Transmission electron microscopy
2DG6P: 2-deoxyglucose-6-phosphate
H&E: Hematoxylin and eosin
DAPI: 2-(4-amidinophenyl)-1 H-indole-6-carboximidamide
OGTT: Oral Glucose Tolerance Test
IPITT: Intraperitoneal Insulin Tolerance Test
BA: Cytometric Bead Array
