# Supplementary material for: Enhancing insulin sensitivity in type 2 diabetes mellitus using apelin-loaded small extracellular vesicles from Wharton’s jelly-derived mesenchymal stem cells: a novel therapeutic approach
Source: Diabetol Metab Syndr. 2024 Apr 16;16:84. doi: 10.1186/s13098-024-01332-w (PMC11020616; doi:10.1186/s13098-024-01332-w)
Supplement: Supplementary file 1 — Supplementary Material 1 [file 13098_2024_1332_MOESM1_ESM.docx]

**Supplementary material**

**Enhancing Insulin Sensitivity in Type 2 diabetes mellitus Using Apelin-Loaded small extracellular vesicles from Wharton's Jelly-Derived mesenchymal stem cells: A Novel Therapeutic Approach**


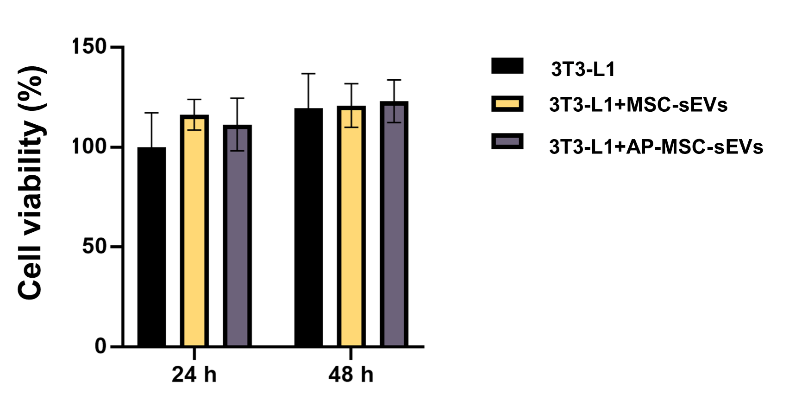


**Figure S1**: Assessment of cell viability in 3T3-L1 cells co-cultured with MSC-sEVs and AP-MSC-sEVs for 24 and 48 hours, as determined by the CCK-8 assay.


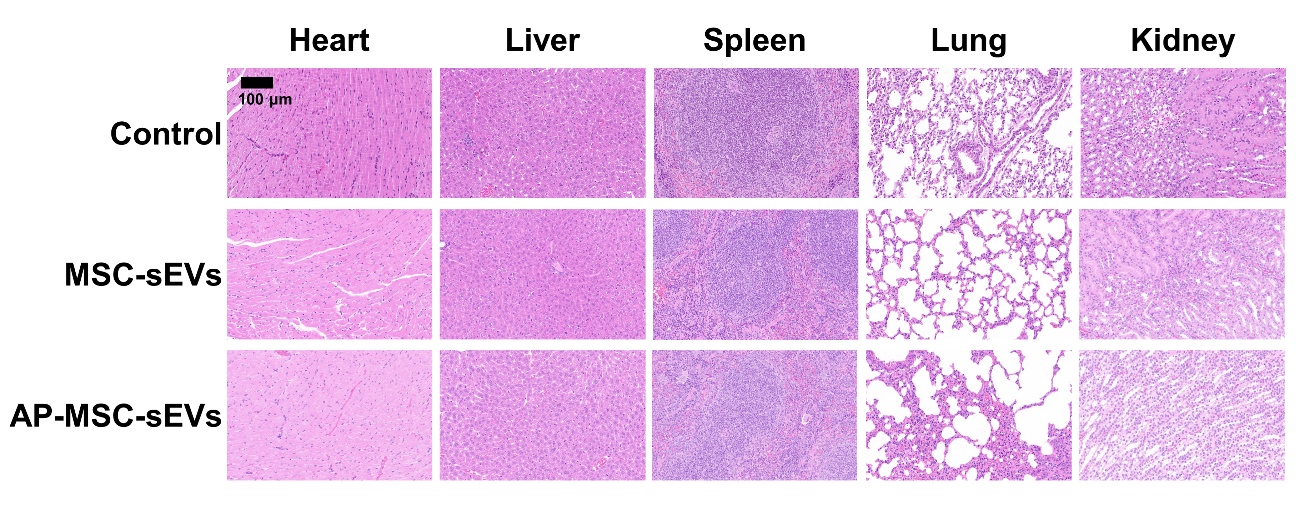


**Figure S2**: H&E staining of various organs in C57 mice three days post-intravenous injection of MSC-sEVs and AP-MSC-sEVs.
